# Multi-site tumor sampling (MSTS) improves the performance of histological detection of intratumor heterogeneity in clear cell renal cell carcinoma (CCRCC)

**DOI:** 10.12688/f1000research.9419.2

**Published:** 2016-09-16

**Authors:** Rosa Guarch, Jesús M. Cortés, Charles H. Lawrie, José I. López

**Affiliations:** 1 Department of Pathology, Complejo Hospitalario B de Navarra, Pamplona, Navarra, 31008, Spain; 2 Quantitative Biomedicine Unit, Biocruces Research Institute, Barakaldo, 48903, Spain; 3 Ikerbasque: The Basque Foundation for Science, Bilbao, 48013, Spain; 4 Department of Cell Biology and Histology, University of the Basque Country (UPV/EHU), Leioa, 48940, Spain; 5 Molecular Oncology Group, Biodonostia Research Institute, San Sebastian, 20014, Spain; 6 Department of Physiology, University of the Basque Country (UPV/EHU), Leioa, 48940, Spain; 7 Radcliffe Department of Medicine, University of Oxford, Oxford, OX3 9DU, UK; 8 Department of Pathology, Cruces University Hospital, University of the Basque Country (UPV/EHU), Barakaldo, 48903, Spain; 9 Biomarkers in Cancer Unit, Biocruces Research Institute, Barakaldo, 48903, Spain

**Keywords:** Intratumor heterogeneity, tumor sampling, multi-site tumor sampling grade, pathology, clear cell renal cell carcinoma

## Abstract

Current standard-of-care tumor sampling protocols for CCRCC (and other cancers) are not efficient at detecting intratumoural heterogeneity (ITH). We have demonstrated *in silico* that an alternative protocol, multi-site tumor sampling (MSTS) based upon the divide and conquer (DAC) algorithm, can significantly increase the efficiency of ITH detection without extra costs. Now we test this protocol on routine hematoxylin-eosin (HE) sections in a series of 38 CCRCC cases. MSTS was found to outperform traditional sampling when detecting either high grade (p=0.0136) or granular/eosinophilic cells (p=0.0114). We therefore propose that MSTS should be used in routine clinical practice.

## Introduction

Clear cell renal cell carcinoma (CCRCC) is the most frequent form of renal cancer in Western Countries ^1^ and a paradigmatic example of intratumoural heterogeneity (ITH) ^2– 5^. ITH is a major factor in the unpredictable clinical behavior and treatment failure response that these tumors can display ^5^ and as a consequence, detection of ITH by pathologists is becoming an increasingly important metric of clinical practice.

We have recently demonstrated that a multi-site tumor sampling (MSTS) protocol following the divide-and-conquer (DAC) algorithm outperforms routine sampling protocols (RS) in detecting ITH when tested *in silico* ^6^. DAC is a well-known strategy in computer science to solve many different practical problems and consists in recursively breaking down a given problem into simpler parts (divide) until they are simple enough to be efficiently solved (conquer). Then, partial solutions are combined to solve the original problem. Since such a strategy does not necessarily increase the cost of procedures and can be performed without significant changes in the pathologist’s routine, we proposed its generalized implementation in pathology labs ^6, 7^. This study extends this hypothesis to a real life scenario by comparing the MSTS protocol with RS when detecting classic morphological ITH in a series of 38 CCRCC.

## Material and methods

**Figure 1.**
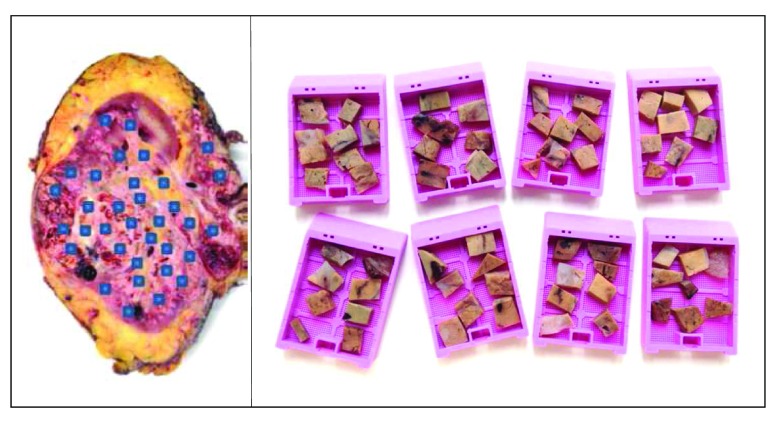
Example of multi-site tumor sampling (MSTS) following the divide and conquer (DAC) strategy.

Thirty-eight CCRCC were prospectively collected from the Pathology Department of the Cruces University Hospital (Barakaldo, Spain). All patients were informed about the potential use for research of their surgically resected tissues, and accepted this eventuality by signing an information consent approved by the local Ethics Committee (CEIC). The two sampling protocols MSTS and RS were applied in each case. The RS ^8^ method consisted of selecting one tissue fragment per centimeter of tumor diameter plus an additional fragment of each suspicious area by the naked eye. Alternatively, the MSTS ^6, 7^ method consisted of selecting a large number of small fragments including six to eight of them in the same cassette and fixing the number of cassettes to one per centimeter of tumor ( Figure 1). Thus, the two sampling protocols made use of the same number of cassettes and, consequently, the total surface of tumor tissue selected for analysis is equivalent (restricted by the cassette dimensions). Tissue samples were fixed in formalin and embedded in paraffin following routine methods. Four-micron-thick histological slides were processed in an automatized stainer (Symphony system, Ventana Medical Systems Inc., Tucson, USA).

The study was performed on hematoxylin-eosin (HE) stained histological slides exclusively. Two experienced pathologists (RG, JIL) reviewed all HE sections in a blind fashion. Fuhrman grade, cell type (clear vs. granular eosinophilic), and the presence of necrosis and/or sarcomatoid change were evaluated in all cases and in both sampling methods. Grade was grouped as low (G1/2) and high (G3/4) for higher consistency. The four histological parameters were considered if they were present in the sample, no matter they were focally or extensively found in RS or affecting one or more fragments in MSTS, since the final goal was “its detection”.

### Statistical analysis

Results of the two methods (RS and MSTS) were compared by applying a chi-squared test (χ ^2^), a test applied to sets of categorical data to evaluate the hypothesis of independence between the two groups. In particular, we made use of the script *chi2test.m* (available to download at http://es.mathworks.com/matlabcentral/fileexchange/16177-chi2test) and run it in Matlab (The Mathworks, Inc, version 2012a). For instance, to test if MSTS detected more high-grade tumors (G3/4) than RS (results in columns H and D respectively from the Excel file containing the raw data), we first counted the total number of *high* labels in RS (column D) and in MSTS (column H), giving a total number of 21 cases for RS and 31 cases for MSTS. Next, considering a total number of 38 CCRCC cases, we run in Matlab *p=chi2test ([31 38-31; 21 38-21])*, which returns a p-value of *p=0.0136*. Similarly, we compare the performances of the two methods with regard to the categories of presence of granular eosinophilic cells, sarcomatoid phenotype and tumor necrosis.

## Results

The series consisted of 32 males and 6 females with an average age of 63 years (range 41–87), and average tumor diameter of 8.5 cm (range 4–15). Overall, MSTS was more informative than RS in 28 of 38 cases (73.5%). In particular, MSTS detected a significantly higher number of high-grade tumors (G3/4) than RS (31 vs. 21 cases respectively, χ ^2^ test, p=0.0136) and a significantly higher number of tumors containing granular eosinophilic cells (32 vs. 22 cases respectively, χ ^2^ test, p=0.0114) ( Table 1).

Although MSTS also detected a higher number of tumors displaying sarcomatoid phenotype (12 vs. 6 cases, respectively) and a higher number of cases presenting tumor necrosis (10 vs. 7 cases, respectively), their figures did not reach significant levels ( Table 1) probably because both were detected by the naked eye and were sampled in both protocols.

**Table 1.** Comparison between both sampling protocols showing that MSTS outperforms RS

Histological parameters	MSTS	RS	p value (χ ^2^ test)
High grade (G3/4)	31	21	0.0136
Granular eosinophilic cells	32	22	0.0114
Sarcomatoid phenotype	12	6	0.1
Tumor necrosis	10	7	0.5

[[i] *MSTS: Multi-site tumor sampling, RS: Routine sampling*]

Moreover, MSTS detected a clear cell papillary renal cell carcinoma (CK7+/CD10-) component in one case that RS missed. 

Clinic-pathological data corresponding to the two RS and MSTS sampling methods in 38 CCRCC Table with the raw data analyzed in the study. The vertical axis shows the cases included in the study (1 to 38). The horizontal axis shows the clinic-pathological parameters analyzed, as follows: A: sex, B: age (years), C. tumour diameter (in centimeters), D to G: Results obtained in the RS protocol (D: tumour grade, E: presence of eosinophilic cells, F: presence of necrosis, and G: presence of sarcomatoid change), H to K: Results obtained in the MSTS protocol (H: tumour grade, I: presence of eosinophilic cells, J: presence of tumour necrosis, and K: presence of sarcomatoid change). A Chi-squared test χ ^2^ was performed between the results obtained in the following paired rows (D and H, E and I, F and J, and G and K) to compare RS and MSTS protocols. Click here for additional data file. Copyright: © 2016 Guarch R et al. 2016 Data associated with the article are available under the terms of the Creative Commons Zero "No rights reserved" data waiver (CC0 1.0 Public domain dedication).

## Discussion

The clinical importance of detecting ITH is becoming clearer as time passes and as a consequence represents one of the most challenging tasks facing pathologists today ^5^. However, pathologists have not yet adapted the old sampling protocols and seem not aware of a concerning paradox: The success of sophisticated devices and expensive platforms in detecting key tumor mutations depends on the selection rightness of tumor pieces which are (very often) made by residents. The combination of lack of solid evidence for the necessity to change current practice and a reluctance to incur new costs and increased work load may be responsible of this attitude.

We present evidence that the MSTS protocol is much more effective than RS in detecting high grade areas and other histological parameters that determine tumor aggressiveness and prognosis in CCRCC. Importantly the MSTS protocol does not incur extra costs to pathology labs ^6, 7^. A similar approach (but for a different purpose) was already reported in 1990 by Battifora and Mehta to optimize the screening of new histologic reagents ^9^.

A thorough histological analysis such as MSTS performs may also help the pathologists in detecting hidden or unexpected tumor histologies, i.e., hybrid tumors, collision neoplasms, histologically complex tumors, and minor but crucial components in a huge tumor, giving definite clues for a complete diagnosis.

However, the final objective of MSTS is to ensure the detection of the complete spectrum of molecular changes across each tumor as this achievement will open new possibilities for more efficient treatments, thus accomplishing oncologists expectancies ^10^. Exhaustive molecular analyses of renal carcinomas are being performed nowadays ^4, 5^, but these approaches are difficult to be implemented in the routine practice for most of the hospitals due to their high costs. In this sense, MSTS could be regarded as an affordable alternative for a generalized application since it takes into account as a necessary premise the sustainability of health systems.

## Data availability

The data referenced by this article are under copyright with the following copyright statement: Copyright: © 2016 Guarch R et al.

Data associated with the article are available under the terms of the Creative Commons Zero "No rights reserved" data waiver (CC0 1.0 Public domain dedication). 

F1000Research: Dataset 1. Clinic-pathological data corresponding to the two RS and MSTS sampling methods in 38 CCRCC, 10.5256/f1000research.9419.d135847 ^11^
