# Supplementary material for: Multi-site tumor sampling (MSTS) improves the performance of histological detection of intratumor heterogeneity in clear cell renal cell carcinoma (CCRCC)
Source: F1000Res. 2016 Sep 16;5:2020. Originally published 2016 Aug 17. [Version 2] doi: 10.12688/f1000research.9419.2 (PMC5007747; doi:10.12688/f1000research.9419.2)
Supplement: Peer review history

# Peer review: Referee response for version 1

**DOI:** 10.5256/f1000research.10144.r15895

**Published:** 2016-09-02

**Authors:** Giuseppe Zamboni, Enrico Munari

**Affiliations:** 1 Department of Pathology, University of Verona, Verona, Italy; 2 Ospedale Sacro Cuore Don Calabria, Negrar, Italy

This is a very interesting work in which the authors applied a simple yet very smart approach to address the very complex issue of intratumoral heterogeneity, for which clear cell renal cell carcinoma stands as a paradigm.

 Such method could indeed be applied to other tumor entities as well; moreover it could form the basis for a practical approach to tackle the problem of the minimum required number of samples that must be collected in order to cover the most of the molecular landscape of tumors.

 Lastly I would like to suggest that the authors might briefly explain in the introduction what is the “divide and conquer” strategy, as they did in their previous paper.

We have read this submission. We believe that we have an appropriate level of expertise to confirm that it is of an acceptable scientific standard.

# Peer review: Referee response for version 1

**DOI:** 10.5256/f1000research.10144.r16008

**Published:** 2016-09-01

**Authors:** Kevin O. Leslie

**Affiliations:** 1 Department of Laboratory Medicine and Pathology, Mayo Clinic Arizona, Phoenix, AZ, USA

This well-designed study details a successful tissue sampling technique for addressing the inherent problem of morphologic diversity present in clear cell renal cell carcinoma. The title is appropriate, the design and methods are sound, and the conclusions are sensible.

I have read this submission. I believe that I have an appropriate level of expertise to confirm that it is of an acceptable scientific standard.

# Peer review: Referee response for version 1

**DOI:** 10.5256/f1000research.10144.r15724

**Published:** 2016-08-30

**Authors:** Miguel A. Piris

**Affiliations:** 1 Pathology Department, Universidad de Cantabria, Santander, Spain

The authors demonstrate that multi-site tumor sampling improves the sensitivity for the detection of molecular heterogeneity in routine paraffin-embeded clear cell renal cell carcinoma samples.

 Molecular heterogeneity is a relevant feature of the advanced cancer samples that determines the adaptation capacity of the tumoral cells and their capacity to survive to the therapy.  Standards for recognizing or reporting tumor heterogeneity are still to be defined. In this sense, this work is an inspiring example. It would be great to know whether this heterogeneity has clinical prognostic or predictive implications.

I have read this submission. I believe that I have an appropriate level of expertise to confirm that it is of an acceptable scientific standard.

# Peer review: Referee response for version 1

**DOI:** 10.5256/f1000research.10144.r15937

**Published:** 2016-08-30

**Authors:** Fabio F. Facchetti

**Affiliations:** 1 Department of Molecular and Translational Medicine, University of Brescia, Brescia, Italy

The study extend previous demonstrations on the usefulness of this original sampling procedure, that might be relevant also to detect variability of molecular landscape in tumors, whatever is their origin.

 Just an annotation to better understand the method used:

- Was the total surface of fragments contained in a MSTS significantly different from that of a RS cassette?
- How was grading and other parameter assigned in the MSTS cassette, in the sense, based even on a single or part of fragment of those contained in the all cassette?
- Similarly, was in the RS given based on part (and how much?) of the section?

I do understand that the procedure used likely followed the published rules, but this might be mentioned in the paper.

I have read this submission. I believe that I have an appropriate level of expertise to confirm that it is of an acceptable scientific standard.

# Peer review: Referee response for version 1

**DOI:** 10.5256/f1000research.10144.r15825

**Published:** 2016-08-23

**Authors:** Jason L. Hornick

**Affiliations:** 1 Department of Pathology, Brigham and Women's Hospital, Harvard Medical School, Boston, MA, USA

Excellent demonstration of the benefits of the proposed sampling approach. Obviously a major goal of such an approach would be to reveal molecular heterogeneity that might be missed by routine sampling. The authors might briefly comment on this in the discussion.

I have read this submission. I believe that I have an appropriate level of expertise to confirm that it is of an acceptable scientific standard.
